# Comparing short to standard duration of antibiotic therapy for patients hospitalized with cellulitis (DANCE): study protocol for a randomized controlled trial

**DOI:** 10.1186/1471-2334-14-235

**Published:** 2014-05-05

**Authors:** Duncan R Cranendonk, Brent C Opmeer, Jan M Prins, W Joost Wiersinga

**Affiliations:** 1 Center for Experimental and Molecular Medicine (CEMM), Center for Infection and Immunity Amsterdam (CINIMA), Academic Medical Center, University of Amsterdam, Meibergdreef 9, room G2-130, 1105 AZ, Amsterdam, The Netherlands; 2 Department of Internal Medicine, Division of Infectious Diseases, Academic Medical Center, 1105 AZ, Amsterdam, The Netherlands; 3 Clinical Research Unit, Academic Medical Center, 1105 AZ, Amsterdam, The Netherlands

**Keywords:** Cellulitis, Erysipelas, Skin infections, Antibiotics, Flucloxacillin, Therapy duration, Hospital setting, Randomised controlled trial

## Abstract

**Background:**

Recommended therapy duration for patients hospitalized with cellulitis is 10–14 days. Unnecessary use of antibiotics is one of the key factors driving resistance. Recent studies have shown that antibiotic therapy for cellulitis in outpatients can safely be shortened, despite residual inflammation. This study will compare in hospitalized patients the safety and effectiveness of shortening antibiotic therapy for cellulitis from 12 to 6 days.

**Methods/design:**

In a multicenter, randomized, double-blind, non-inferiority trial, adult patients admitted with cellulitis will be included. Cellulitis is defined as warmth, erythema, and induration of the skin and/or subcutaneous tissue, with or without pain (including erysipelas). All patients will initially be treated with intravenous flucloxacillin, and will be evaluated after 5–6 days. Those who have improved substantially (defined as being afebrile, and having a lower cellulitis severity score) will be randomized at day 6 between additional 6 days of oral flucloxacillin (n = 198) or placebo (n = 198). Treatment success is defined as resolution of cellulitis on day 14 (disappearance of warmth and tenderness, improvement of erythema and edema), without the need of additional antibiotics for cellulitis by day 28. Secondary endpoints are relapse rate (up to day 90), speed of recovery (using a cellulitis severity score until day 28, and VAS scores on pain and swelling until day 90), quality of life (using the SF-36 and EQ-5D questionnaires) and costs (associated with total antibiotic use and health-care resource utilization up to day 90).

**Discussion:**

Inclusion is planned to start in Q2 2014.

**Trial registration:**

ClinicalTrials.gov (NCT02032654) and the Netherlands Trial Register (NTR4360).

## Background

Cellulitis is an acute, spreading pyogenic inflammation of the skin (dermis) and subcutaneous tissue, usually complicating a wound, ulcer or dermatosis [1]. Erysipelas, while originally considered a separate disease entity with a well-defined raised edge and more prominent systemic symptoms, is now considered one of the manifestations of cellulitis, owing to physicians often not being able to make the clinical distinction, and the similar spectrum of causative agents [2-4]. Cellulitis is among the most frequent infections leading to hospitalization [5]. In the USA, hospital visits for cellulitis and abscesses increased from 17.3 to 32.5 per 1000 person-years between 1997–2010, resulting in 600.000 hospitalizations in 2010 [6]. Similarly, the combined incidence of cellulitis and erysipelas of the leg (CEL) in the Netherlands increased from 1.7 to 22 per 1000 persons-years between 2001–2007, of which 5-10% were hospitalized. This increase is probably due to a rise in both the elderly population, and the number of patients with diabetes or other causes of immune suppression [7,8]. Relapse rates are around 12% after 21 days to a year [9-11], and up to 14-29% after three years [11-13].

As of yet, there is no consensus on admission criteria or rating scales of severity [14,15]. Patients admitted to the hospital for cellulitis generally receive their initial antibiotic regimen intravenously (IV). After initial IV therapy, switching to oral antibiotics is generally accepted when patients are afebrile, inflammatory symptoms are regressing and inflammatory markers are improving [16,17]. As most cellulitis cases are caused by streptococci and *Staphylococcus aureus*, Dutch national guidelines advise 10–14 days of flucloxacillin for patients with cellulitis (dosage: oral 500 mg q.d.s. or IV 1000 mg q.d.s.) [18]. This takes into account that methicillin-resistant *Staphylococcus aureus* (MRSA) prevalence in the Netherlands is still well below 5%, while it is an increasing problem in many parts of the world [19]. The Infectious Diseases Society of America (IDSA) recommends a beta-lactam for non-purulent cellulitis, MRSA targeting antibiotics for purulent cellulitis, and treatment durations between 5–10 days for outpatients and 7–14 days for inpatients [20]. However, due to a lack of evidence the recommended duration of therapy in both of these guidelines is mainly based on expert opinion [2].

It is postulated that, like persisting radiological abnormalities in pneumonia [21], erythema that persists after a few days of antibiotic treatment is due to residual inflammation. If this is indeed the case, this would allow physicians to discharge patients or end antibiotic treatment before full symptom resolution [22,23]. A recent study reported that 5 days of antibiotics is not inferior to 10 days of antibiotics in outpatients with uncomplicated cellulitis responding to initial therapy, despite residual symptoms on day 5 [23]. In line, studies on urinary tract infections, respiratory tract infections, and other infectious diseases have also demonstrated that antibiotic therapy can safely significantly be shortened [24-31]. In light of these studies, we hypothesize that for patients hospitalized with cellulitis, treatment with a short course (6 days) of antibiotics is not inferior to a standard course (12 days), if they respond to initial treatment.

## Methods/design

The study is registered in the Netherlands Trial Register (NTR4360) and on ClinicalTrials.gov (NCT02032654). The publication on main study results will be in accordance with the Consolidated Standards of Reporting Trials (CONSORT) statement [32].

### Design and sites

The DANCE trial is an investigator-initiated, prospective, multicenter, randomized, investigator-, caregiver- and patient blinded non-inferiority trial, with two parallel groups with an allocation ratio of 1:1. Subjects will initially be enrolled at eight Dutch hospitals: i) Academic Medical Center, Amsterdam; ii) VU University Medical Center, Amsterdam; iii) University Medical Center, Utrecht; iv) Diakonessenhuis, Utrecht; v) Flevoziekenhuis, Almere; vi) Onze Lieve Vrouwe Gasthuis, Amsterdam; vii) Sint Lucas Andreas Hospital, Amsterdam; viii) Slotervaart Hospital, Amsterdam. These sites serve a large, densely populated urban area of the Netherlands. In the case of slow enrolment, additional hospitals will be approached to participate in the study. This study will be conducted in accordance with the Declaration of Helsinki and was approved by the medical ethics committee of the Academic Medical Center, Amsterdam. The Boards of Directors of all participating sites subsequently approved the execution of the study in their centers.

### Participants

Adult patients meeting the in- but not exclusion criteria are eligible (see below). Cellulitis is defined as warmth, erythema, and induration of the skin and/or subcutaneous tissue, with or without pain (including erysipelas) [33]. Eligible patients are approached by local investigators who will provide them with the patient information folder. Two investigators (DRC and a research nurse) will be responsible for the informed consent process and data collection in every site. They will perform all study procedures. If this proves to be excessively time consuming, additional investigators will be recruited and trained. After providing consent, subjects can leave the study at any time for any reason if they wish to do so, without any consequences. The investigator can decide to withdraw a subject from the study for urgent medical reasons. Once randomized, withdrawn subjects will not be replaced, in accordance with the intention-to-treat principle.

The inclusion criteria are:

•Admitted to receive intravenous antibiotics for cellulitis/erysipelas

•18 years of age or older

•Capable of giving written informed consent, able to comply with study requirements and restrictions

The exclusion criteria are:

•Allergy for flucloxacillin, other beta-lactam antibiotics or one of the additives, or history of flucloxacillin-induced hepatitis or liver enzyme disorders.

•Concurrent use of antibiotics for other indications

•Alternative diagnosis accounting for the clinical presentation.

•All cases involving any of the following factors:

o Use of antibiotics with Gram-positive activity for more than 4 days in the past 7 days

o Intensive care unit admission during the last 7 days

o Severe peripheral arterial disease (Fontaine IV)

o Severe cellulitis necessitating surgical debridement or fascial biopsy

o Necrotizing fasciitis

o Periorbital or perirectal involvement

o Surgery

o Life expectancy less than one month

o Risk factors associated with Gram-negative pathogens as a causative agent [34]:

▪ Chronic or macerated infra-malleolar ulcers, or infra-malleolar ulcers with previous antibiotic treatment, in patients with diabetes mellitus [35].

▪ Neutropenia

▪ Cirrhosis (Child-Pugh class B or C)

▪ Intravenous drug use

### Procedures

All participants will receive regular antibiotics (flucloxacillin, or another empiric beta-lactam with activity against *S. aureus* that is shortly after replaced by flucloxacillin) for the first 6 days of treatment. Treatment will be started intravenously, but a switch to oral medication can be performed at the caregiver’s discretion when the patient has become afebrile and the cellulitis shows improvement. Participants will be assessed by one of the investigators at multiple time points (Table 1).

**Table 1:** Study assessments, questionnaires and specimen collections

	**Visit 1**	**Visit 2**	**Visit 3**	**Visit 4**	**Visit 5**	**Visit 6**
	**Day 1**	**Day 2-3**	**Day 5-6**	**Day 14**	**Day 28**	**Day 90**
**Assessment**						
Informed consent	X					
In-/Exclusion criteria	X					
Baseline characteristics	X					
Randomization criteria		X	X			
Randomization of patients eligible for randomization			X			
Physical examination	X		X	X	X	
Cellulitis severity score	X	X	X	X	X	
VAS	X	X	X	X	X	X
Medication adherence				X		
Non-study medication	Updated throughout study					
Lab and culture results	Updated throughout study					
**Questionnaires**						
SF-36	X				X	X
EQ-5D	X				X	X
HCRUQ			X		X	X
**Specimen collection**						
Blood samples	X	X	X	X	X	
Skin biopsy*	X					
Skin swabs	X			X		

VAS = visual analog scales on pain and swelling; SF-36 = Short Form (36) questionnaire; EQ-5D = EuroQol-5D questionnaire; HCRUQ = health-care resource utilisation questionnaire. *only performed in patients admitted to the main study site.

Participants responding to initial therapy, defined as absence of fever (temperature > 38.0°C) and improvement in cellulitis severity score by day 5–6 compared to the first 24 hours of admission, and able to take oral medication will be randomized into one of two groups. One group will receive 6 more days of flucloxacillin (500 mg q.d.s.), while the other group will receive 6 days of placebo (500 mg q.d.s.). Of note, patients with a creatinine clearance of <10 ml/min or those on haemodialysis will receive 500 mg of trial medication t.d.s. Patients are not randomized if: i) they require ICU admission, concomitant antibiotics, or surgical intervention, or develop an allergic reaction (see exclusion criteria); ii) their blood cultures become positive (excluding contamination); or iii) they do not improve. Other aspects of treatment are according to standard of care. Compression therapy should be encouraged, but is not mandatory, and is at the discretion of the caregiver. Patients will be informed about the correct use of the trial medication. To register adherence, patients will be asked to bring left-over trial medication to their day 14 visit, at which the capsules will be counted and afterwards destroyed.

### Assessments

Standard demographic and other patient characteristics will be collected, including age, gender, non-trial medication use, comorbidity, ethnic background, recreational drug use, smoking, body weight, residential status, and risk factors for cellulitis [6] (i.e. venous insufficiency, lymphoedema, peripheral arterial disease, immunosuppression, diabetes, trauma, tattoos, ulcers, eczema, tinea pedis and burns). The extent of cellulitis (length and width, lesion surface area) and presence of lymphadenopathy will be recorded on pre-determined time points (Table 1). A cellulitis severity score will be used, which measures erythema, warmth, tenderness, edema, ulceration, drainage and fluctuation. Each parameter is scored with a numerical value of 0–3 (none, mild, moderate, and severe, respectively). This has been used previously in a trial on duration of therapy for uncomplicated cellulitis [23].

The two investigators responsible for data collection will standardize these assessments by performing simultaneous assessment at the beginning of the study. When five patients have been scored independently with identical scores, the investigators will start assessing patients on their own. After 100 patients have been assessed, the investigators will again assess five patients together. VAS scores and questionnaires will be collected through a web survey tool. Microbiological culture results and hematology and chemistry lab results will be collected from regular care, when available. Additionally, blood samples will be taken at multiple time points, and skin biopsies and swabs will be taken from selected patients on admission.

### Outcome measures

The primary outcome is resolution of cellulitis at day 14, defined as disappearance of warmth and tenderness at the site of infection, with substantial improvement in erythema and edema, and without recurrence by day 28, defined as the need for additional antibiotic therapy for cellulitis.

Secondary outcomes are recurrence of cellulitis by day 90; speed of recovery, determined by improvement in cellulitis severity score and through self-assessment of subjective pain and swelling on visual analog scales (VAS) from 0–10; mean health-related quality of life, using the SF-36 and EQ-5D questionnaires; and costs, associated with total antibiotic use and other health-care resource utilisation, measured with modified versions of iMTA’s Productivity Cost Questionnaire (iPCQ) and Medical Consumption Questionnaire (iMCQ).

Blood samples and skin biopsies will be used for biomarker discovery, fundamental research, and skin microbiota analyses.

### Randomization and blinding

Patients eligible for randomization will be allocated to either (1) flucloxacillin or (2) placebo in a 1:1 randomization ratio, using the online ALEA® software developed by the NKI-AVL (Amsterdam, NL). This independent central randomization service will create a computer generated random schedule of blocks with random block sizes (maximum block size of 6), stratified for presence of diabetes mellitus and study site. Upon randomization, the software will generate a randomization number for the investigators, and will send an automated e-mail to the appropriate pharmacy with the randomization number and randomization result. Pharmacies will dispense randomly numbered trial medication containers to the patients, depending on the randomization result, working from a confidential list which describes the content (flucloxacillin or placebo) of each numbered container. Flucloxacillin and placebo are in similar capsules, and are administered in the same dosage and duration, leading to blinding of patients, investigators and caregivers. The randomization code will only be broken in case the Data Safety Monitoring Board (DSMB) advises to do so.

### Sample size

When the sample size in each group is 198 (396 total), a two-group large-sample normal approximation test with a one-sided 0.050 significance level will have 80% power to reject the null-hypothesis that 6 days of antibiotics is not inferior to 12 days of antibiotics. This assumes a conservatively estimated treatment failure rate of 20%, and considers a 10% absolute difference in treatment failure rate between the two groups as equivalence limit.

### Analysis plan

Statistical analysis will be performed using the modified intention-to-treat principle (mITT), including all randomized patients who received at least one dose of the study medication. Missing data will be handled with multiple imputation. Baseline assessments and outcome parameters will be summarized. Continuous variables will be summarized with standard descriptive statistics, categorical variables with frequencies and percentages.

For the primary outcome, the proportion of successful treatments will be calculated for the two groups, and the absolute risk difference (with one sided 95% confidence limit) will be estimated to evaluate the non-inferiority hypothesis (Table 2). In a secondary analysis, the difference in proportion in the two groups will be adjusted for relevant baseline covariates, using a logistic regression analysis. The difference in proportion of successful treatments will be estimated at mean values for the covariates. Differences in secondary outcome parameters will be reported as relative risk or mean difference (with 95% confidence interval), and p-values for statistical significance estimated with the Chi-square test, t-test or Mann–Whitney test, where appropriate. A Kaplan-Meier analysis will be performed to compare the time to relapse (defined as requiring additional antibiotics for cellulitis) between the two groups.

**Table 2:** Statistical analysis plan

**Variable/outcome**	**Hypothesis**	**Outcome measure**	**Methods of analysis**
**1. Primary**			
Resolution without relapse	Intervention not inferior to control	Percent cured on day 14 without relapse by day 28 [binary]	Absolute risk difference
**2. Secondary**			
Relapse by day 90	No difference	Percent with relapse [binary]	Chi^2^ ➔ Relative risk
Time to relapse	No difference	Relapse after cure [time to event]	Kaplan-Meier survival analysis
Objective speed of recovery	No difference	Cellulitis severity score [continuous]	Student’s T-test or Mann–Whitney U test
Subjective speed of recovery	No difference	Visual analogue scales of pain and swelling [continuous]	Student’s T-test or Mann–Whitney U test
Quality of life	No difference	SF-36 questionnaire score at day 1, 28 and 90 [continuous]	Student’s T-test or Mann–Whitney U test
Additional antibiotic usage	No difference	Total amount of additional antibiotic use, in DDD [continuous]	Student’s T-test or Mann–Whitney U test
Health care utilization	No difference	Total treatment associated costs, Health Care Utilization Questionnaire [continuous]	Student’s T-test or Mann–Whitney U test
**3. Subgroup analyses**			
Cellulitis severity score on a continuous scale	Severity score affects cure rate		Regression analysis, with interaction term for severity score
Diabetes mellitus vs no diabetes mellitus	Diabetes affects cure rate		Regression analysis, with interaction term for (no) diabetes
**4. Sensitivity analyses**	Intervention not inferior to control	All outcomes	
Per protocol analysis			Chi^2^ ➔ Relative risk
Primary outcome adjusted for covariates			Logistic regression analysis

A per protocol analysis will also be performed, for this analysis the following randomized patients of whom the compliance is clear will be included: patients with treatment failure who have received at least 24 hours of study medication, and patients with treatment success who have received at least four days of study medication.

For this analysis treatment success is defined as resolution of cellulitis at 14 days – disappearance of warmth and tenderness at the site of infection, with substantial improvement in erythema and edema –without recurrence by day 28, defined as the need of additional antibiotic therapy for cellulitis [23]. Treatment failure is defined as the persistence or progression of signs and symptoms of the acute process at 14 days after randomization, a recurrence before day 28, or the inability to complete the study owing to adverse events. The response is deemed indeterminate when the patient (i) received less than 80% of the study drug for reasons other than treatment failure, (ii) acquired a concomitant infection outside of the skin requiring antibiotic treatment, (iii) was lost to follow-up, or (iv) died unrelated to the primary diagnosis.

Two subgroup analyses are planned, and statistically tested with interaction effects: one for patients with diabetes mellitus, and one based on the cellulitis severity score. Diabetes mellitus is known to influence outcome in patients treated for complicated skin and skin structure infections, through multiple suggested mechanisms [36]. A high cellulitis severity score reflects an extensive local inflammatory response, which might indicate a higher grade of infection that possibly requires more intensive or prolonged antibiotic therapy.

### Data safety monitoring board

An independent DSMB has been established, consisting of two infectious disease specialists and a methodologist. They will evaluate mortality after 198 patients have completed the trial. Given the expected low mortality rates [37], the power to detect differences in mortality at this stage will be very low. Therefore, the DSMB will qualitatively evaluate all deaths for their possible relation with the given treatment. Only in case of striking differences between the two arms the DSMB will inform investigators and the medical ethics committee. The frequency of other DSMB meetings will depend on the study proceedings and the judgment of the DSMB chair.

## Discussion

It is important that antibiotic exposure is kept to a minimum without compromising patient safety, in order to minimize the emergence of antibiotic resistance [38]. Unnecessary usage of antibiotics, like prolonged treatment duration, is one of the key factors driving resistance [39]. Ideally, the most narrow-spectrum antibiotic is used for the shortest period of time. The problem is that antibiotic treatment duration studies, especially for cellulitis, are relatively scarce amidst the plethora of clinical trials that usually compare newer to older antibiotics, sometimes with different durations. Only one trial has been published that was specifically designed to examine treatment duration in patients with cellulitis; however that one was performed in an outpatient setting [23]. This study will be the first to investigate the optimal antibiotic treatment duration for cellulitis in hospitalized patients, a group of patients that has increased significantly over the last few years and is responsible for the bulk of the cellulitis associated health care expenditure.

One of the current study protocol’s strong points is that the in- and exclusion criteria allow for a large group of cellulitis patients to be included, while still excluding accepted confounders, making the results applicable to a large population. Randomizing at day 5–6 allows for better implementation of the results into clinical practice, since response to initial therapy will be the main determinant when deciding on cessation or continuation of treatment. The short time period in which the primary outcome is measured will hopefully lead to a minimal loss to follow-up. Another strong point is data accuracy, as we will limit the number of main investigators who will assess the outcome parameters, in order to reduce inter-observer variability. Photographs of selected patients will be used to check the accuracy of the measured cellulitis severity scores.

An important challenge will be patient recruitment, as in many randomized controlled trials. We will evaluate the recruitment rate at multiple time points, and if it is too low, additional centers will be approached to assist in inclusion. This should not interfere with data quality, as the same investigators will continue to perform the study procedures, despite the increasing number of participating centers. Attention will also be given to compliance, as trial medication is one capsule size larger than regular flucloxacillin, possibly leading to decreased compliance and thus bias towards non-inferiority. This will be, at least partially, mitigated by careful explanation of the necessity of taking trial medication upon entrance into the trial, and performing pill-counts at the end of the study.

If this trial shows non-inferiority of short course antibiotic therapy in cellulitis, this would allow for systematically shortening therapy for the entire spectrum of skin and soft tissue diseases [23].

### Trial status

Patient recruitment for the trial will start from the beginning of 2014. Data collection is estimated to finish in 2017.

### Finances

This study is financed by a grant of the Netherlands Organisation for Health Research and Development (ZonMW) (grant nr 836011024).

## Abbreviations

DANCE: Duration of ANtibiotic therapy for CEllulitis; MRSA: Methicillin resistant *Staphylococcus aureus*; VAS: Visual analog scales on pain and swelling; SF-36: Short Form (36) questionnaire; EQ-5D: EuroQol-5D questionnaire; HCRUQ: Health-care resource utilisation questionnaire; DSMB: Data safety monitoring board.

## Competing interests

The authors declare that they have no competing interests.

## Authors’ contributions

DC and WJW conceived the study and drafted the manuscript. BCO and JP participated in designing the study and assisted in drafting the manuscript. WJW obtained funding for the study. All authors have read the final manuscript, and give approval for it to be published.

## Pre-publication history

The pre-publication history for this paper can be accessed here:

http://www.biomedcentral.com/1471-2334/14/235/prepub

## References

[B1] Swartz MN Cellulitis N Engl J Med 2004 14 904 912 10.1056/NEJMcp031807 14985488

[B2] Kilburn S Featherstone P Higgins B Brindle R Interventions for cellulitis and erysipelas Cochrane Database Syst Rev 2010 14 1 64 10.1002/14651858.CD004299.pub2 PMC8693180 20556757

[B3] Chira S Miller LG Staphylococcus aureus is the most common identified cause of cellulitis: a systematic review Epidemiol Infect 2010 14 313 317 10.1017/S0950268809990483 19646308

[B4] Gunderson CG Martinello RA A systematic review of bacteremias in cellulitis and erysipelas J Infect 2012 14 148 155 10.1016/j.jinf.2011.11.004 22101078

[B5] Christensen KLY Holman RC Steiner CA Sejvar JJ Stoll BJ Schonberger LB Infectious disease hospitalizations in the United States Clin Infect Dis 2009 14 1025 1035 10.1086/605562 19708796

[B6] Phoenix G Das S Joshi M Diagnosis and management of cellulitis Br Med J 2012 14 e4955 10.1136/bmj.e4955 22872711

[B7] Goettsch WG Bouwes Bavinck JN Herings RMC Burden of illness of bacterial cellulitis and erysipelas of the leg in the Netherlands J Eur Acad Dermatol Venereol 2006 14 834 839 16898907 10.1111/j.1468-3083.2006.01657.x

[B8] Wielink G Koning S Oosterhout R Wetzels R Nijman F Draijer L Dutch College of General Practitioners Guideline on bacterial skin infections Huisarts Wet 2007 14 426 444

[B9] Bergkvist PI Sjöbeck K Relapse of erysipelas following treatment with prednisolone or placebo in addition to antibiotics: a 1-year follow-up Scand J Infect Dis 1998 14 206 207 10.1080/003655498750003708 9730318

[B10] Jenkins TC Sabel AL Sarcone EE Price CS Mehler PS Burman WJ Skin and soft-tissue infections requiring hospitalization at an academic medical center: opportunities for antimicrobial stewardship Clin Infect Dis 2010 14 895 903 10.1086/656431 20839951

[B11] Ellis Simonsen SM van Orman ER Hatch BE Jones SS Gren LH Hegmann KT Lyon JL Cellulitis incidence in a defined population Epidemiol Infect 2006 14 293 299 16490133 10.1017/S095026880500484X PMC2870381

[B12] Cox NH Colver GB Paterson WD Management and morbidity of cellulitis of the leg J R Soc Med 1998 14 634 637 10730111 10.1177/014107689809101206 PMC1296982

[B13] Jorup-Rönström C Britton S Recurrent erysipelas: predisposing factors and costs of prophylaxis Infection 1987 14 105 106 10.1007/BF01650206 3110071

[B14] Perelló-Alzamora M-R Santos-Duran J-C Sánchez-Barba M Cañueto J Marcos M Unamuno P Clinical and epidemiological characteristics of adult patients hospitalized for erysipelas and cellulitis Eur J Clin Microbiol Infect Dis 2012 14 2147 2152 10.1007/s10096-012-1549-2 22298240

[B15] Morris AD Cellulitis and erysipelas Clin Evid (Online) 2008 14 1 8 PMC2907977 19450336

[B16] Stevens DL Eron LL Cellulitis and soft-tissue infections Ann Intern Med 2009 14 ITC11 19124814 10.7326/0003-4819-150-1-200901060-01001

[B17] Clinical Resource Efficiency Support Team (CREST) Guidelines on the Management of Cellulitis in Adults 2005 Belfast, Ireland

[B18] Conceptrichtlijn cellulitis en erysipelas van de onderste extremiteiten [Concept guideline cellulitis and erysipelas of the lower extremities] [http://www.huidziekten.nl/richtlijnen/conceptrichtlijn-cellulitis-en-erysipelas-2013.pdf]

[B19] Antimicrobial resistance interactive database [http://www.ecdc.europa.eu/en/healthtopics/antimicrobial_resistance/database/Pages/database.aspx]

[B20] Liu C Bayer A Cosgrove SE Daum RS Fridkin SK Gorwitz RJ Kaplan SL Karchmer AW Levine DP Murray BE Rybak MJ Talan DA Chambers HF Clinical practice guidelines by the infectious diseases society of america for the treatment of methicillin-resistant Staphylococcus aureus infections in adults and children Clin Infect Dis 2011 14 e18 e55 10.1093/cid/ciq146 21208910

[B21] Bruns AHW Oosterheert JJ El Moussaoui R Opmeer BC Hoepelman AIM Prins JM Pneumonia recovery: discrepancies in perspectives of the radiologist, physician and patient J Gen Intern Med 2010 14 203 206 10.1007/s11606-009-1182-7 19967464 PMC2839328

[B22] Eron LJ Lipsky BA Low DE Nathwani D Tice AD Volturo GA Managing skin and soft tissue infections: expert panel recommendations on key decision points J Antimicrob Chemother 2003 14 Suppl 1 i3 i17 14662806 10.1093/jac/dkg466

[B23] Hepburn MJ Dooley DP Skidmore PJ Ellis MW Starnes WF Hasewinkle WC Comparison of short-course (5 days) and standard (10 days) treatment for uncomplicated cellulitis Arch Intern Med 2004 14 1669 1674 10.1001/archinte.164.15.1669 15302637

[B24] Milo G Katchman E Paul M Christiaens T Baerheim A Leibovici L Duration of antibacterial treatment for uncomplicated urinary tract infection in women Cochrane Database Syst Rev 2009 14 1 81 10.1002/14651858.CD004682.pub2 PMC12700497 15846726

[B25] Michael M Hodson EM Craig JC Martin S Moyer V Short versus standard duration oral antibiotic therapy for acute urinary tract infection in children Cochrane Database Syst Rev 2010 14 1 29 10.1002/14651858.CD003966 12535494

[B26] Sandberg T Skoog G Hermansson AB Kahlmeter G Kuylenstierna N Lannergård A Otto G Settergren B Ekman GS Ciprofloxacin for 7 days versus 14 days in women with acute pyelonephritis: a randomised, open-label and double-blind, placebo-controlled, non-inferiority trial Lancet 2012 14 484 490 10.1016/S0140-6736(12)60608-4 22726802

[B27] Falagas ME Karageorgopoulos DE Grammatikos AP Matthaiou DK Effectiveness and safety of short vs. long duration of antibiotic therapy for acute bacterial sinusitis: a meta-analysis of randomized trials Br J Clin Pharmacol 2009 14 161 171 10.1111/j.1365-2125.2008.03306.x 19154447 PMC2670373

[B28] Altamimi S Khalil A Khalaiwi KA Milner RA Pusic MV Al Othman MA Short-term late-generation antibiotics versus longer term penicillin for acute streptococcal pharyngitis in children Cochrane Database Syst Rev 2012 14 1 56 10.1002/14651858.CD004872.pub3 PMC11984625 22895944

[B29] Pugh R Grant C Cooke RPD Dempsey G Short-course versus prolonged-course antibiotic therapy for hospital-acquired pneumonia in critically ill adults Cochrane Database Syst Rev 2012 14 72

[B30] el Moussaoui R de Borgie CA van den Broek P Hustinx WN Bresser P van den Berk GE Poley JW van den Berg B Krouwels FH Bonten MJM Weenink C Bossuyt PM Speelman P Opmeer BC Prins JM Effectiveness of discontinuing antibiotic treatment after three days versus eight days in mild to moderate-severe community acquired pneumonia: randomised, double blind study BMJ 2006 14 1355 10.1136/bmj.332.7554.1355 16763247 PMC1479094

[B31] Havey TC Fowler RA Daneman N Duration of antibiotic therapy for bacteremia: a systematic review and meta-analysis Crit Care 2011 14 R267 10.1186/cc10545 22085732 PMC3388653

[B32] Schulz KF Altman DG Moher D CONSORT 2010 Statement: Updated guidelines for reporting parallel group randomised trials J Clin Epidemiol 2010 14 834 840 10.1016/j.jclinepi.2010.02.005 20346629

[B33] Calandra GB Norden C Nelson JD Mader JT Evaluation of New Anti-Infective Drugs for the Treatment of Selected Infections of the Skin and Skin Structure Clin Infect Dis 1992 14 Suppl 1 S148 54 1477222 10.1093/clind/15.supplement_1.s148

[B34] Gunderson CG Cellulitis: definition, etiology, and clinical features Am J Med 2011 14 1113 1122 10.1016/j.amjmed.2011.06.028 22014791

[B35] Lipsky BA Berendt AR Deery HG Embil JM Joseph WS Karchmer AW LeFrock JL Lew DP Mader JT Norden C Tan JS Diagnosis and treatment of diabetic foot infections Clin Infect Dis 2004 14 885 910 10.1086/424846 15472838

[B36] Lipsky BA Itani KMF Weigelt JA Joseph W Paap CM Reisman A Myers DE Huang DB The role of diabetes mellitus in the treatment of skin and skin structure infections caused by methicillin-resistant Staphylococcus aureus: results from three randomized controlled trials Int J Infect Dis 2011 14 e140 e146 10.1016/j.ijid.2010.10.003 21134775

[B37] Garau J Ostermann H Medina J Avila M McBride K Blasi F Current management of patients hospitalized with complicated skin and soft tissue infections across Europe (2010–2011): assessment of clinical practice patterns and real-life effectiveness of antibiotics from the REACH study Clin Microbiol Infect 2013 14 e377 e385 10.1111/1469-0691.12235 23663184

[B38] Laxminarayan R Duse A Wattal C Zaidi AKM Wertheim HFL Sumpradit N Vlieghe E Hara GL Gould IM Goossens H Greko C So AD Bigdeli M Tomson G Woodhouse W Ombaka E Peralta AQ Qamar FN Mir F Kariuki S Bhutta ZA Coates A Bergstrom R Wright GD Brown ED Cars O Antibiotic resistance-the need for global solutions Lancet Infect Dis 2013 14 1057 1098 10.1016/S1473-3099(13)70318-9 24252483

[B39] Niederman MS Antimicrobial Agents Principles of appropriate antibiotic use Antimicrob Agents 2005 14 170 175 10.1016/s0924-8579(05)80324-3 16543079

